# Supplementary material for: NRF2 deficiency increases obesity susceptibility in a mouse menopausal model
Source: PLoS One. 2020 Feb 11;15(2):e0228559. doi: 10.1371/journal.pone.0228559 (PMC7012419; doi:10.1371/journal.pone.0228559)
Supplement: S1 Table — (DOCX) [file pone.0228559.s002.docx]

**Supplemental Information**

**Supplemental Table 1. Quality control parameters of biochemical and ELISA assays**

| Assay | Range | Sensitivity | Intra CV | Inter CV | Min sample |
| --- | --- | --- | --- | --- | --- |
| GLU | 1.4-28 mmol/l | >1.4 mmol/l | <5% | <5% | 2 ul |
| T-CHO | 0-10.34 mmol/l | >1.0 mmol/l | <3% | <5% | 2 ul |
| TG | 0-9.04 mmol/l | >1.0 mmol/l | <5% | <8% | 2 ul |
| LDL | 0.2-12 mmol/l | >0.2 mmol/l | <8% | <10% | 2 ul |
| HDL | 0.065-3.8 mmol/l | >0.065 mmol/l | <3% | <5% | 2 ul |
| E2 | 2-64 pmol/ml | >0.1 pmol | <10% | <15% | 10 ul |
| MDA | 24.69-2000 ng/ml | <8.84 ng/ml | <10% | <12% | 25 ul |
| 5-HT | 7.5-240 ng/ml | >7.5 ng/ml | <10% | <15% | 25 ul |
| DA | 3.75-120 pg/ml | >3.75 pg/mol | <10% | <15% | 25 ul |
