# Supplementary material for: NRF2 deficiency increases obesity susceptibility in a mouse menopausal model
Source: PLoS One. 2020 Feb 11;15(2):e0228559. doi: 10.1371/journal.pone.0228559 (PMC7012419; doi:10.1371/journal.pone.0228559)
Supplement: Attachment — Submitted filename: Gangula review.docx [file pone.0228559.s003.docx]

The article entitled “Nrf2 deficiency increases obesity susceptibility in a mouse menopausal model” explains the importance of Nrf2 on metabolic abnormalities in menopausal women using overiectomized mouse menopausal model. The authors show the importance of Nrf2 gene in onset of obesity, which is already known with several published articles. Obesity itself has shown to reduce Nrf2 expression, as well as Nrf2 deletion alters estrus cycle, which is validated in nrf2^-/-^ mice. The mechanistic aspects of Nrf2 deletion on menopause is lacking. The manuscript needs some revisions,

1. The authors need to add the references for estradiol levels, estrus cycle in Nrf2 deletion.
2. It is known that overiectomy is done in female mice, but the authors need to mention the mouse gender used in this study?
3. In the methodology the authors don’t mention time points study but in the results section they have results at different time points (Fig. 4). This needs to be explained in the methodology.
4. The collection of blood through retro-orbital (eyelid blood collection) which is mentioned in the methods is for end point or terminal experiments. Did the authors collect blood through this method at the end of each time point? If not how much of the serum did they use for the detection of glucose and lipid metabolism indices at each time point?
5. Standard deviation in most of the results is more than the mean values? (Fig. 1, Fig. 3 & Fig. 4)
6. Fig. 3b, change in body weights significance between the groups needs to be shown?
7. In the discussion, there is a sentence which says “ numerous studies have shown that estrogen……” but only one reference quoted?
